# Supplementary material for: Causal Relationship between Aging and Anorexia Nervosa: A White-Matter-Microstructure-Mediated Mendelian Randomization Analysis
Source: Biomedicines. 2024 Aug 16;12(8):1874. doi: 10.3390/biomedicines12081874 (PMC11351342; doi:10.3390/biomedicines12081874)
Supplement: Supplementary file 1 [file biomedicines-12-01874-s001.zip › Supplementary Figure/Figure S4/Singe SNP plot of MR analysis for TL to ubm-b-1978.pdf]

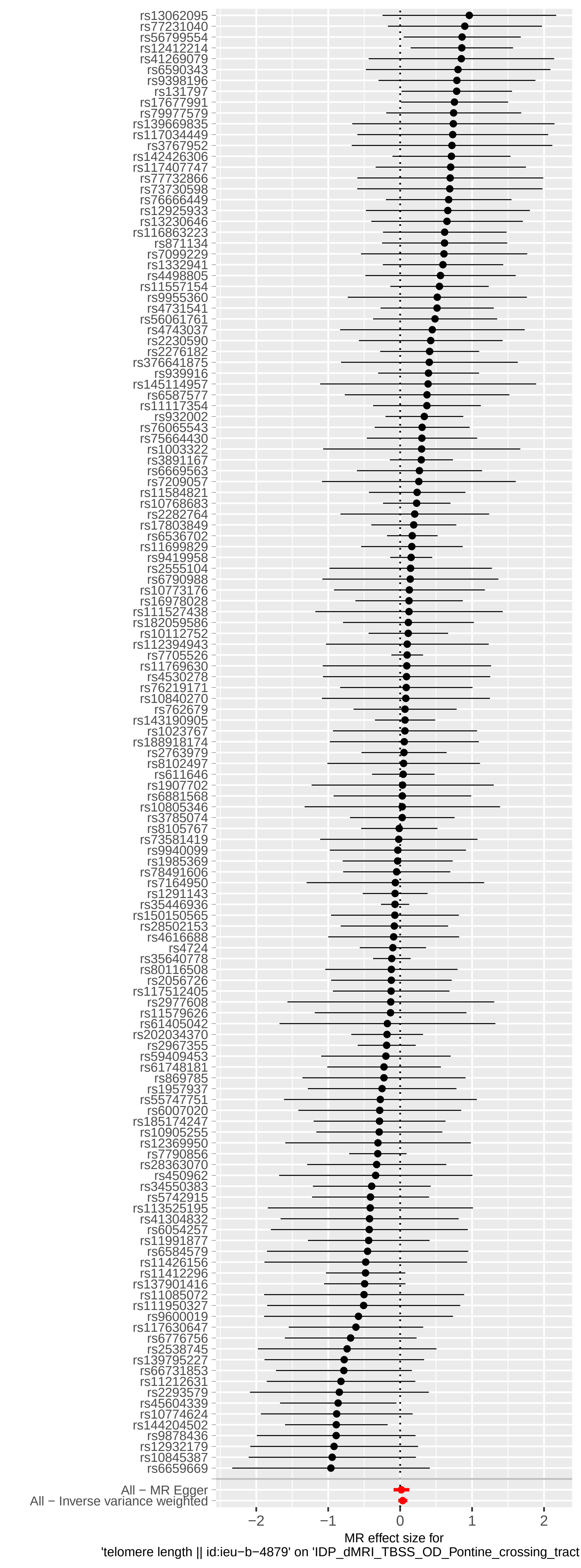
rs13062095
rs77231040
rs56799554
rs12412214
rs41269079
rs6590343
rs9398196
rs131797
rs17677991
rs79977579
rs139669835
rs117034449
rs3767952
rs142426306
rs117407747
rs77732866
rs73730598
rs76666449
rs12925933
rs13230646
rs116863223
rs871134
rs7099229
rs1332941
rs4498805
rs11557154
rs9955360
rs4731541
rs56061761
rs4743037
rs2230590
rs2276182
rs376641875
rs939916
rs145114957
rs6587577
rs11117354
rs932002
rs76065543
rs75664430
rs1003322
rs3891167
rs6669563
rs7209057
rs11584821
rs10768683
rs2282764
rs17803849
rs6536702
rs11699829
rs9419958
rs2555104
rs6790988
rs10773176
rs16978028
rs111527438
rs182059586
rs10112752
rs112394943
rs7705526
rs11769630
rs4530278
rs76219171
rs10840270
rs762679
rs143190905
rs1023767
rs188918174
rs2763979
rs8102497
rs611646
rs1907702
rs6881568
rs10805346
rs3785074
rs8105767
rs73581419
rs9940099
rs1985369
rs78491606
rs7164950
rs1291143
rs35446936
rs150150565
rs28502153
rs4616688
rs4724
rs35640778
rs80116508
rs2056726
rs117512405
rs2977608
rs11579626
rs61405042
rs202034370
rs2967355
rs59409453
rs61748181
rs869785
rs1957937
rs55747751
rs6007020
rs185174247
rs10905255
rs12369950
rs7790856
rs28363070
rs450962
rs34550383
rs5742915
rs113525195
rs41304832
rs6054257
rs11991877
rs6584579
rs11426156
rs11412296
rs137901416
rs11085072
rs111950327
rs9600019
rs117630647
rs6776756
rs2538745
rs139795227
rs66731853
rs11212631
rs2293579
rs45604339
rs10774624
rs144204502
rs9878436
rs12932179
rs10845387
rs6659669
All − MR Egger
All − Inverse variance weighted
−2
−1
0
1
2
MR effect size for
'telomere length || id:ieu−b−4879' on 'IDP_dMRI_TBSS_OD_Pontine_crossing_tract
